# The structure, function and expression analysis of the nodulin 26-like intrinsic protein subfamily of plant aquaporins in tomato

**DOI:** 10.1038/s41598-022-13195-0

**Published:** 2022-06-02

**Authors:** Yuxiang Zhang, Shihong Fei, Yunmin Xu, Yong He, Zhujun Zhu, Yuanyuan Liu

**Affiliations:** Collaborative Innovation Center for Efficient and Green Production of Agriculture in Mountainous Areas of Zhejiang Province, College of Horticulture Science, Zhejiang A&F University, Hangzhou, 311300 Zhejiang China

**Keywords:** Molecular biology, Plant sciences

## Abstract

The nodulin 26-like intrinsic protein (NIP) family belonging to a group of aquaporin proteins is unique to plants. NIPs have a wide of transport activities and are involved in developmental processes and stress tolerance. The well reported Lsi1 and Lsi6 belonging to NIP III were characterized as Si transporters. However, except Lsi1 and Lsi6, most NIPs remain unknown. Here, we identified 43 putative aquaporins in tomato. We found there are 12 NIPs, including 8 NIP I proteins, 3 NIP II proteins, and 1 NIP III protein among the 43 aquaporins. Also, there are two Si efflux transporters SlLsi2-1 and SlLsi2-2 identified by using Lsi2 proteins from other species. By analysing the phylogenetic relationships, conserved residues and expression patterns, we propose that three NIP I members (SlNIP-2, SlNIP-3 and SlNIP-11) may transport water, ammonia, urea, and boric acid, and contribute to pollen development. Three NIP II proteins (SlNIP-7, SlNIP-9 and SlNIP-12) may be boric acid facilitators, and affect plant growth and anther development. Overall, the study provides valuable candidates of Si transporters and other NIP proteins to further explore their roles in uptake and transport for silicon, boron, and other substrates in tomato.

## Introduction

Silicon (Si) is a common element, and its abundance in the earth's crust is second only to oxygen, reaching 28.8%^1^. Si is an essential element for animals and is closely related to animal life activities^2^. Although Si is not a vital element for plants^1,3^, a large number of studies have shown that Si has beneficial effects on plant growth and development, especially under stressed conditions, such as drought, salt, heavy metal, high temperature, radiation damage, freezing, nutrient imbalance^4–6^. In plants, Si has been proposed as a quasi-essential element, and the International Plant Nutrition Institute (IPNI) defined Si as a "beneficial substance" in 2015. The main forms of Si existing in soil are silica (SiO_2_) and silicate, and only a small part exists in the form of silicic acid Si(OH)_4_^7^, which is the only form of Si that can be absorbed by plants^8^.

Although Si is found in most plants^7^, the content of Si varies greatly among different plants^9^. According to the differences in Si absorption modes, plants were classified into active, passive and rejective Si absorption plants^10^. In addition, plants are divided into three types: high Si accumulators, intermediate Si accumulators and low Si accumulators^3^. Monocots such as rice and wheat belong to high Si accumulation plants and active Si absorption plants, and their Si content can reach 1.0–10.0% of dry weight. Most of the eudicots, such as cucumbers, belong to intermediary Si accumulation plants and passive Si absorption plants, and the Si content in their bodies is about 0.2–1.0% of dry weight. Some of the eudicots, such as tomato, belong to low Si accumulation plants and Si rejected plants, and their Si content is only less than 0.2% of dry weight^11^.

The absorption of Si by plants is achieved mainly by two steps. Firstly, silicic acid is absorbed by roots, and then silicic acid is transported to aboveground by transpiration pull^12^. Among them, although transpiration pull can promote the transfer of Si in plants from roots to shoots, studies have proved that transpiration pull is not the reason for the difference of Si accumulation in different plants, but the uptake ability of root plays a major role in Si uptake and transport in plants^12^. The uptake process of Si is mediated by specific transmembrane proteins, Low silicon rice 1 (Lsi1), identified as Si transporters. The first identified Si transporter in higher plants is OsLsi1 in rice^13^. OsLsi1 is located in the distal side of the plasma membrane of the exodermis and endodermis cells. Its function is to facilitate Si uptake into cells of exodermis from soil solution, and promote Si uptake into cells of endodermis from aerenchyma^13^. Shortly after the discovery of OsLsi1, a second Si transporter gene, OsLsi2, was uncovered^14^. OsLsi2 is located in the proximal side of the plasma membrane of the exodermis and endodermis cells. Its function is to facilitate Si efflux into aerenchyma from exodermis cells and facilitate Si efflux into stele from endodermis cells^14^. Obviously, OsLsi1 is an influx transporter, while OsLsi2 is an efflux transporter^13,14^. A third Si transporter identified as influx transporter is OsLsi6 in rice^15^. OsLsi6 is primarily located in the parenchyma cells of xylem in the leaf sheaths and leaves, which is responsible for xylem unloading and further distribution of Si in aerial tissues^15,16^.

It has been reported that Lsi1 and Lsi6 belong to the Nodulin 26-like intrinsic proteins (NIPs) subfamily of the aquaporin (AQP) family^13^, while Lsi2 falls into the ion transporter superfamily^14^. AQPs are water channel proteins, also called major intrinsic proteins (MIPs) that facilitate the transport of water and a variety of solutes, such as boric acid, NH4^+^, glycerol or urea^17^. Based on the sequence similarity and the localization, plant AQPs can be divided into five groups. Except NIPs, the other four groups include the plasma membrane intrinsic proteins (PIPs), the tonoplast intrinsic proteins (TIPs), the small basic intrinsic proteins (SIPs), and the X intrinsic proteins (XIPs)^18–21^. Among these AQPs, NIPs are unique to plants. Furthermore, NIPs can be classified into three subgroups—NIP I, NIP II and NIP III—with respect to the composition of the aromatic/arginine (ar/R) selectivity filter which is formed by four residues from helices 2 (H2), H5, loop E (LE1) and LE2^22,23^. The size and chemical properties of the residues in ar/R selectivity filter may determine the size and chemical properties of substrates NIPs can transport. NIP I can transport water, glycerol^24^ and lactic acid^25^; NIP II can transport boron^26^ and urea^27^; whereas, NIP III have transport activities for silicon, arsenic and selenium^22^. Indeed, unlike AQP1 belonging to NIP I which contains two bulky amino acid residues in the H2/H5, OsLsi1 belonging to NIP III contains two small residues has a broader pore diameter of the channel^28^. Therefore, OsLsi1 can transport relatively larger molecules of silicic acid (4.38 Å), while the substitution of larger Phe and His for smaller Gly and Ser at H2 and H5 constrains the AQP1 pore to 2.8 Å permitting the flux of water (diameter 2.4 Å) and the exclusion of bulkier solutes^23,28^.

Si is extremely low in tomato plant. At present, there is still a lack of research on the Si uptake and transport in tomato, and the reasons for its low Si accumulation are still controversial. One study first reported that SlLsi1 with 109-AA instead of 108-AA spacing between NPA domains showed loss of Si permeability, compared with the functional Lsi1 transporters in high Si-accumulators^29^. The other study believed that SlLsi1 is normally functional, but loss-of-function of SlLsi2 cannot transfer silicic acid absorbed from roots to xylem, thus resulting in low Si accumulation in shoot^30^. Although tomato is a Si excluder, Si could benefit tomato a lot on many aspects, such as seed germination, shoot growth, the root/shoot ratio, osmotic adjustment, photosynthetic rate, hydraulic conductance, transpiration, antioxidant systems^31^. Evidences on Si transporters in tomato, even in eudicots, are relatively insufficient. The functions of other NIP proteins other than SlLsi1 in tomato are also still unknown. In the present study, Lsi1 and Lsi6 Si influx transporters and Lsi2 Si efflux transporters from other species were set to blast tomato homologues. 43 aquaporin proteins homologous to Lsi1 and Lsi6, and 2 ion transporter proteins homologous to Lsi2 were identified in tomato. Detailed information, including their phylogenetic relationships, gene structures, conserved domains, subcellular localizations, tissue expression profiles, and cis-acting elements were analyzed. The studies will provide a solid basis for further functional characterization of tomato Si transporters as well as other NIP members, and exploring molecular mechanism of their uptake and transport for silicon, boron, and other substrates in tomato.

## Results

### Identification of Lsi1/Lsi2/Lsi6 homologues of aquaporin and ion subfamily in tomato

Tomato Si transporter homologues were identified from the tomato genome database (SGN, https://solgenomics.net) by a BLAST search using the full-length protein sequences of reported Si transporters Lsi1/Lsi2/Lsi6 from rice (*Oryza sativa* L.) and cucumber (*Cucumis sativus* L.). The amino acid sequences of Lsi1/Lsi2/Lsi6 from rice and cucumber were downloaded from NCBI database (Supplementary Table S1). A total of 43 tomato aquaporin proteins and 2 ion transporter proteins were obtained after deletion of repetitive sequences.

### Phylogenetic analysis and classification of tomato Lsi1/Lsi2/Lsi6 homologues

**Figure 1:**
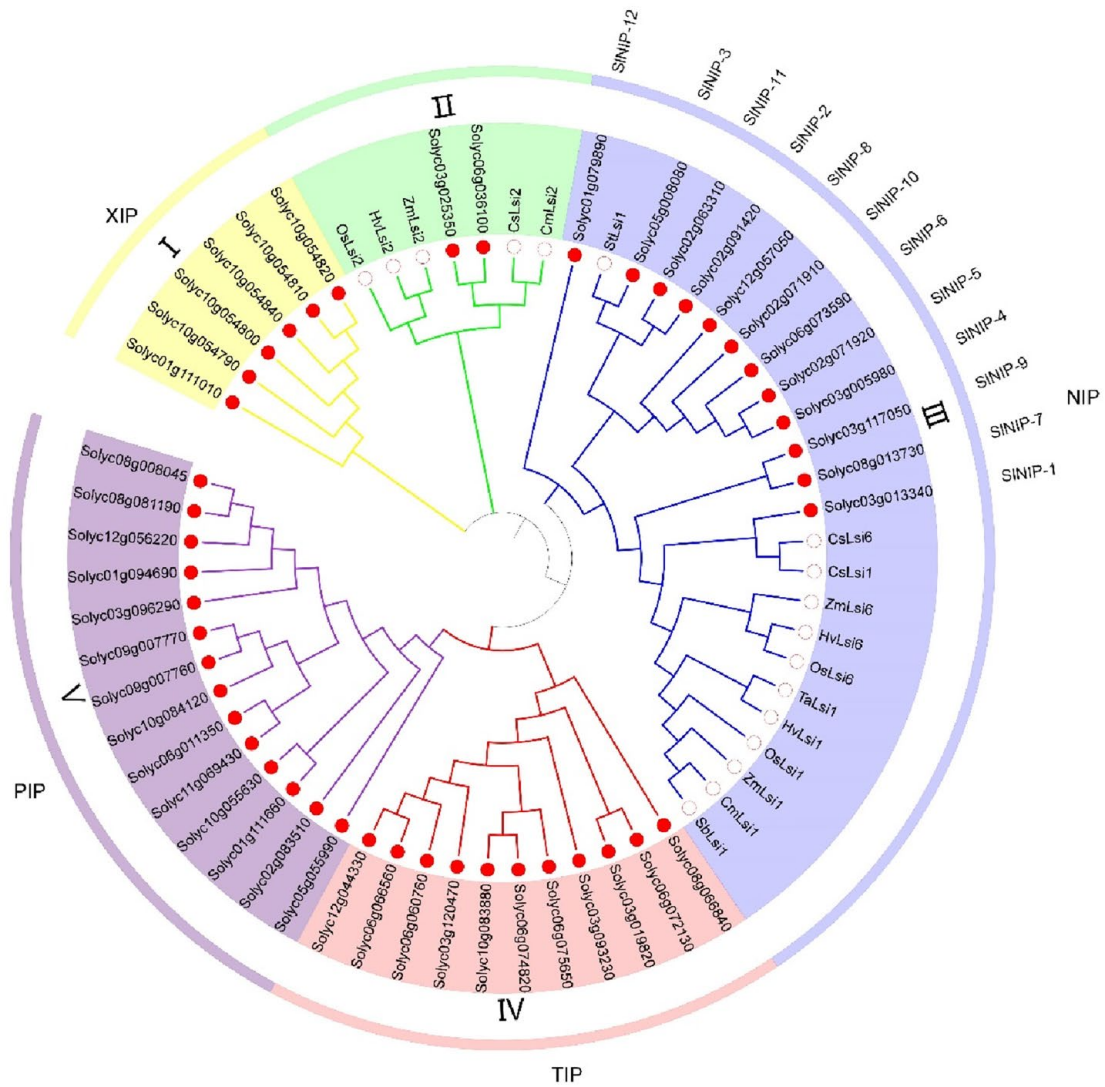
Phylogenetic analysis of 45 homologous proteins of Si transporters identified in tomato and Si transporters available in other species. The tree was constructed using the maximum likelihood method (MLM) with 1000 bootstraps. Each of the five different clades is represented by a specific color and named as I, II, III, IV and V. Proteins from tomato are marked with red solid circles. Proteins from other species are marked with hollow circles. *Hv* stands for barley (*Hordeum vulgare* L.), *Ta* stands for wheat (*Triticum aestivum* L.), *Zm* stands for corn (*Zea mays* L.), *Sb* stands for sorghum (*Sorghum bicolor* L.), *Cm* stands for pumpkin (*Cucurbita moschata* L.), *St* stands for potato (*Solanum tuberosum* L.), *Os* stands for rice (*Oryza sativa* L.), *Cs* stands for cucumber (*Cucumis sativus* L.).

To reveal the phylogenetic relationships of 45 tomato homologues, the amino acid sequences of reported 17 Lsi1/Lsi2/Lsi6 Si transporters from other species were included (Supplementary Table S1) for the construction of phylogenetic tree. These 62 proteins can be classified into five clades (I–V, Fig. 1). Notably, each clade contains tomato homologues, six are assigned to Clade I. Two are classified into Clade II that includes reported Lsi2 Si transporters. Twelve are members of Clade III, together with the well reported Lsi1/Lsi6 Si transporters. Eleven are divided into Clade IV, and fourteen belong to Clade V. Actually, members in Clade I, Clade III, Clade IV and Clade V correspond to members in XIPs, NIPs, TIPs, and PIPs described in current literature^32^. Twelve tomato homologues were grouped in Clade III with StLsi1 of potato, CsLsi1 and CsLsi6 of cucumber, ZmLsi1 and ZmLsi6 of maize, HvLsi1 and HvLsi6 of barley, OsLsi1 and OsLsi6 of rice, TaLsi1 of wheat, CmLsi1 of pumpkin, SbLsi1 of sorghum. It has been reported that OsLsi1^13^, OsLsi6^15^, HvLsi1^33^, ZmLsi1 and ZmLsi6^34^, CsLsi1^35^ belong to NIP III subfamily of aquaporins, suggesting the twelve tomato homologues in Clade III also belong to the NIP subgroup. We name them as SlNIP-1 (Solyc03g013340), SlNIP-2 (Solyc02g091420), SlNIP-3 (Solyc05g008080), SlNIP-4 (Solyc03g005980), SlNIP-5 (Solyc02g071920), SlNIP-6 (Solyc06g073590), SlNIP-7 (Solyc08g013730), SlNIP-8 (Solyc12g057050), SlNIP-9 (Solyc03g117050), SlNIP-10 (Solyc02g071910), SlNIP-11 (Solyc02g063310) and SlNIP-12 (Solyc01g079890), respectively. Solyc03g025350 and Solyc06g036100 clustered in Clade II with OsLsi2 of rice, HvLsi2 of barley, ZmLsi2 of maize, CsLsi2 of cucumber and CmLsi2 of pumpkin. Based on protein sequence similarity and their relationship in the phylogenetic tree, the two orthologous Lsi2-like proteins of tomato in Clade II were named as SlLsi2-1 (Solyc06g036100) and SlLsi2-2 (Solyc03g025350) (Supplementary Table S2).

Lsi1 and Lsi6 in other species have been identified as Si influx transporters, and Lsi2 has been characterized as Si efflux transporters. In Lsi1/Lsi6 proteins-containing Clade III, the orthologous SlNIP-1 was the most homologous to these Si influx transporters, and reasonably predicted to have Si influx transport activity. Similarly, the orthologous SlLsi2-1 and SlLsi2-2 were proposed to have efflux transport activity for Si. However, in a previous study, no Si transport activity of SlLsi1 (designated SlNIP-1 in this study) was detected in *Xenopus* oocytes^29^. This report is not consistent with the other study which determined that SlLsi1 was a functional influx transporter^30^. SlLsi2-L1 (designated SlLsi2-1 in this study) and SlLsi2-L2 (designated SlLsi2-2 in this study) were reported no efflux transport activity for Si^30^. However, the function of aquaporins in tomato remain to be largely uncharacterized, except SlNIP-1, members in Clade III that belong to NIP subgroup are still unknown.

### The chromosomal localization of 45 Lsi1/Lsi2/Lsi6 homologous genes in tomato

**Figure 2:**
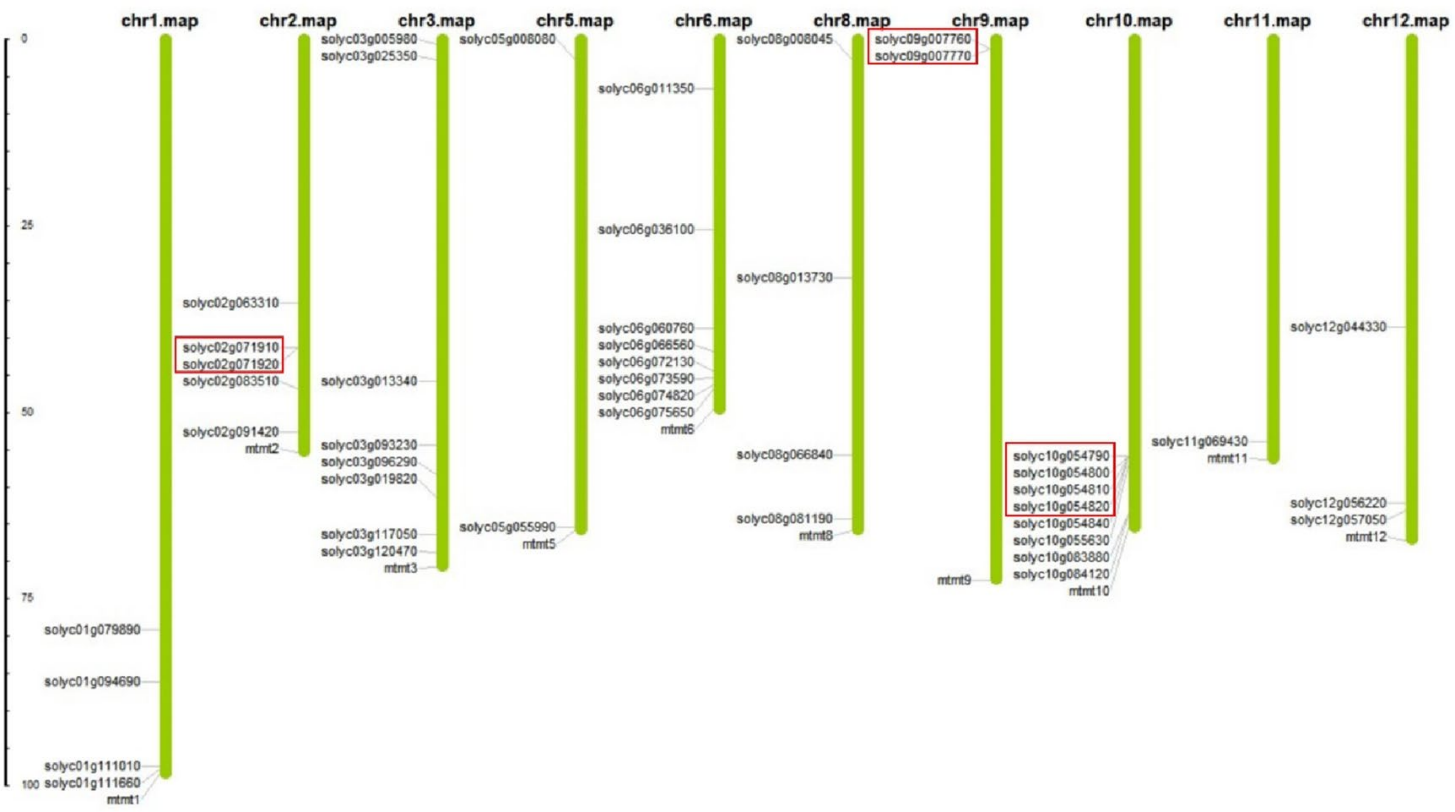
Distribution of homologous genes of Si transporters on tomato linkage groups (LGs). The 45 homologous genes of Si transporters were mapped onto 10 LGs in the tomato genome. The LG number was shown on the top of each LG. The rule on the left indicates the physical map distance among genes (Mbp). The red boxs indicate tandem array genes.

The chromosomal localizations of 45 tomato Lsi1/Lsi2/Lsi6 homologues were determined to visualize their genomic position information. They were distributed on 10 of 12 chromosomes except for chromosome 4 and chromosome 7 (Fig. 2). Among them, eight genes were mapped to chromosome 3, 6 and 10; two on chromosome 5 and 9, three on chromosome 12, four on chromosome 1 and 8, five on chromosome 2; there was only one gene on chromosome 11 (Fig. 2). It should be noted that gene duplication may occur among these tomato Si transporter homologues, since the loci Solyc10g054790, Solyc10g054800, Solyc10g054810 to Solyc10g054820 are found next to each other on chromosome 10. Also the gene pairs Solyc02g071910/Solyc02g071920, Solyc09g007760/Solyc09g007770 were found on chromosome 2 and chromosome 9, respectively. Interestingly, Solyc10g054790, Solyc10g054800, Solyc10g054810 and Solyc10g054820 were clustered together in Clade I, Solyc02g071910/Solyc02g071920 were grouped together in Clade III, and Solyc09g007760/Solyc09g007770 were clustered together in Clade V (Fig. 1), which showed high similarity based on protein sequences, indicating they may share common functions with each other.

### Analysis of exon–intron structure

The exon and intron information of 45 tomato Lsi1/Lsi2/Lsi6 homologues were searched in SGN database and sorted by GSDS. The color bar on the left side of the gene name represents the clades of these genes in the evolutionary tree. Exon–intron analysis showed that the size and the number of the exons are highly conserved within each clade, but significantly different among the clades. Most members of the 45 tomato Lsi1/Lsi2/Lsi6 homologous genes contained three to five exons, but a few contained less than three or more than five exons. Members of Clade I and Clade II are characterized by three exons. Most members of Clade III have five exons. The majority of the members of Clade IV features three exons, while most members of Clade V contain four exons (Supplementary Fig. S1A,B).

### Gene structure and motif composition analysis

Motifs are highly conserved amino acid residues in homologous proteins, which may play important roles in the structure and function of active proteins. The conserved motifs of these 45 tomato Lsi1/Lsi2/Lsi6 homologues were analyzed by MEME. Ten conserved motifs were identified (Supplementary Fig. S1C). Among them, Motif 9 only exists in the members of Clade II and this clade does not contain any other motifs, which might contribute to the functional divergence of Lsi2 proteins in Clade II. It is reasonable Lsi2 was ion transporter protein, differing from NIP proteins. Motifs 1, 2 and 3 were found in Clade III that clustered by Lsi1/Lsi6 proteins, which were also shared in Clade I, IV and V. Interestingly, members of Clade I and V, and some members of Clade IV, but not any members of Clade III, also shared Motif 7. Motif 8 exists in all members of Clade V and some members of Clade I; in addition, members in Clade I solely contain Motifs 5, 6 and 10, and members in Clade V uniquely possess Motif 4 (Supplementary Fig. S1A,C). These observations suggest genes in Clade I, IV and V may have evolved from genes in Clade III, sharing common motifs (Motifs 1, 2 and 3) and similar functions with members in Clade III, and further gained some additional motifs (such as Motif 7) and corresponding functional diversification afterwards.

### Evolutionary analysis of NIP subfamily from tomato, Arabidopsis and rice

**Figure 3:**
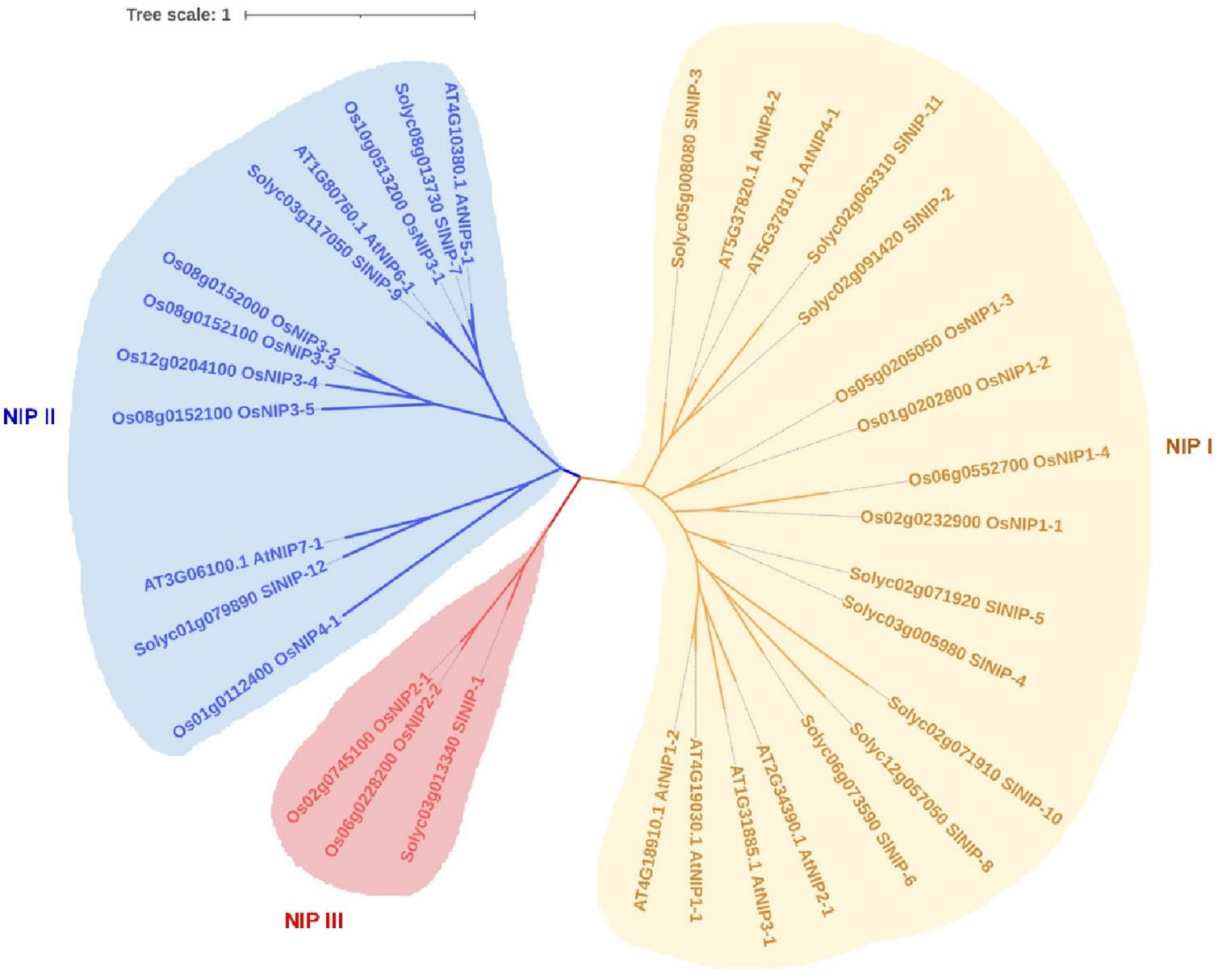
Evolutionary analysis of twelve, nine, twelve NIP proteins from tomato, *Arabidopsis* and rice. The polygenetic tree was constructed by MEGAX using maximum likelihood method, and was modified by iTOL. Members in NIP I, II and III were shown by red, blue and yellow colors, respectively.

The well-characterized Lsi1 channels belong to the NIP subfamily of aquaporins. In our study, we have found 12 members that in Clade III belong to the NIP subgroup (Fig. 1). However, except SlNIP-1, the other members in NIP subgroup are still unknown. Therefore, we have made an in-depth study on these twelve NIP proteins. To well understand their role in substrate selectivity, we constructed phylogenetic analyses of these 12 NIPs with orthologues from *Arabidopsis*^36^ and rice^37^ (Fig. 3), the accession numbers of the corresponding proteins were listed in Supplementary Table S3. As phylogenetic tree showed that the *Arabidopsis* and rice NIPs were distributed in two (I and II) and three (I, II and III) subgroups, respectively, which is well consistent with previous reports^29^. The 12 tomato NIPs fall into three NIP subgroups (Fig. 3). SlNIP-1 is grouped with well-defined OsNIP2-1 (OsLsi1) and OsNIP2-2 (OsLsi6) that belong to NIP III subgroup. SlNIP-7, SlNIP-9 and SlNIP-12 fall into NIP II subgroup, and were tightly clustered together with AtNIP5-1, AtNIP6-1 and AtNIP7-1, respectively. Eight out of the twelve tomato NIPs belong to NIP I, including SlNIP-2, SlNIP-3, SlNIP-4, SlNIP-5, SlNIP-6, SlNIP-8, SlNIP-10 and SlNIP-11. Among them, SlNIP-2, SlNIP-3 and SlNIP-11 showed relatively closed to AtNIP4-1 and AtNIP4-2, whereas SlNIP-4, SlNIP-5, SlNIP-6, SlNBIP-8 and SlNIP-10 showed highly closed to AtNIP1-1, AtNIP1-2, AtNIP2-1, and AtNIP3-1 (Fig. 3).

### Prediction of physicochemical properties and identification of conserved domain

**Figure 4:**
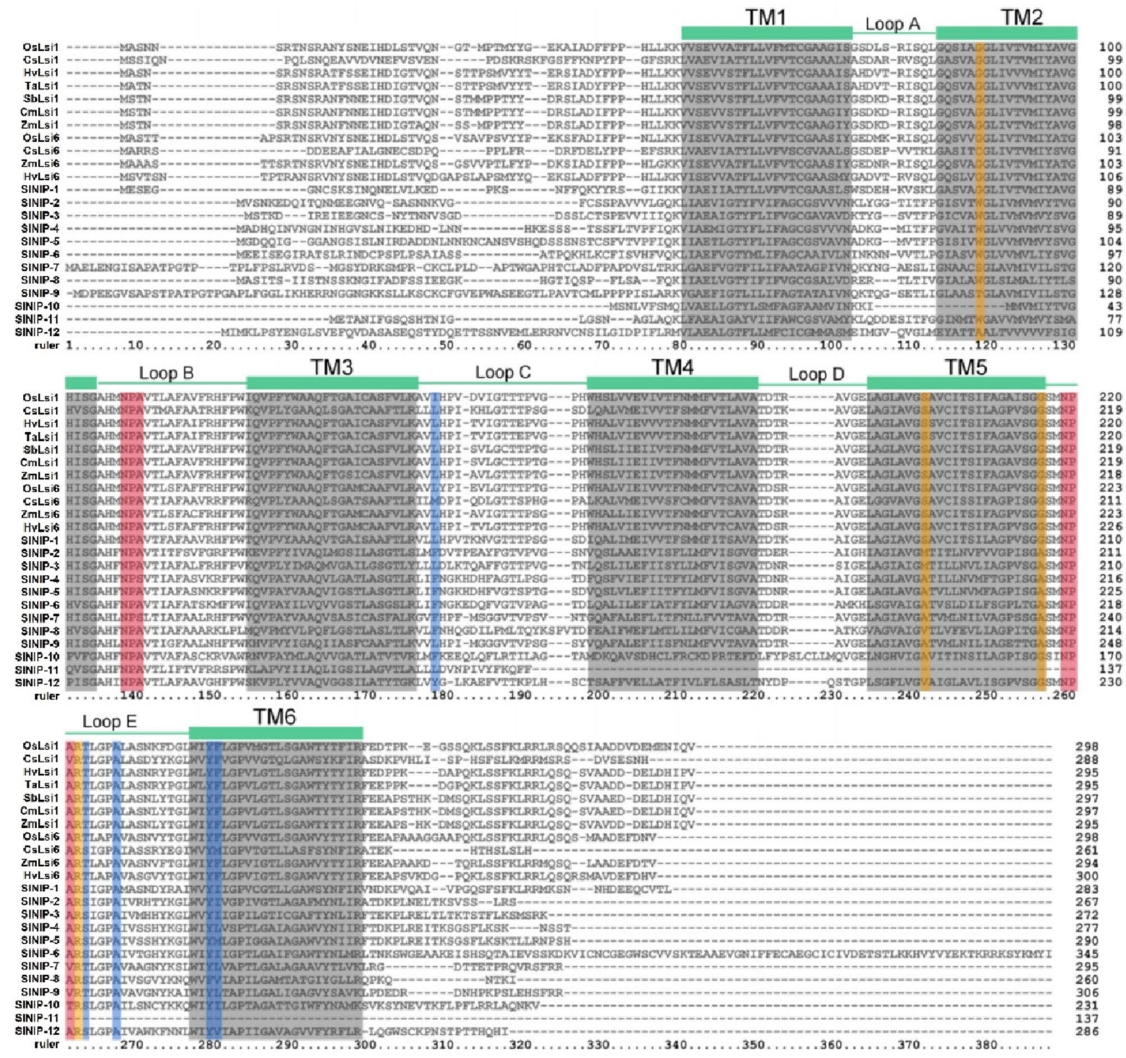
Identification of conserved domains of Si transporter Lsi1/6 homologues (SlNIP-1 to SlNIP-12) in tomato and Si transporters Lsi1/6 reported in other plants. Protein sequence alignment of functionally known Si transporters Lsi1/6 in other species and the newly identified homologues in tomato. The gray region represents the transmembrane (TM) domains, the red region represents the conserved NPA domains, the yellow region represents the ar/R selectivity filter sites, and the blue region represents the Froger’s residue sites.

It is well known that NIPs of aquaporin subfamily were characterized for six transmembrane domains (TMDs) connected by five loops (loop A—loop E)^38^, two highly conserved NPA (asparagine-proline-alanine) motifs on loop B and loop E, an ar/R selectivity filter and Froger’s positions^39^. Sequence alignments of twelve tomato NIPs were performed with ClustalX2 (Fig. 4). The TMDs of each protein were predicted by TMHMM2.0 online tool, the positions of TMDs are marked with gray areas, and the numbers of TMDs of each protein are recorded in Table 1. All the identified Si transporters of Lsi1/Lsi6 in other species contain six TMDs, and most of the twelve tomato NIPs also contain six TMDs. However, there is an exception for SlNIP-10 and SlNIP-11, which possess four and three TMDs, respectively. The second TMD in SlNIP-10 is incomplete, while SlNIP-11 missed the fourth, fifth and sixth TMDs (Table 1).

**Table 1:** Characteristics of tomato homologues of Si transporters, and analysis of their protein information, subcellular localization, conserved amino acid residues, conserved motifs and transmembrane domains (TMDs).

Name	Len	MW	pI	GR	TM	ar/R selectivity filter				Froger residue					NPA motif			LOC
						H2	H5	LE1	LE2	P1	P2	P3	P4	P5	LB	LE	DB	
SlNIP-1	283	30.08	8.76	0.37	6	G	S	G	R	L	S	A	Y	I	NPA	NPA	109	plas
SlNIP-2	267	28.27	7.78	0.66	6	W	V	A	R	F	S	A	Y	I	NPA	NPA	109	plas
SlNIP-3	272	29.27	8.24	0.66	6	W	V	A	R	L	S	A	Y	I	NPA	NPA	109	plas
SlNIP-4	277	29.66	9.16	0.43	6	W	V	A	R	F	S	A	Y	L	NPS	NPA	109	plas
SlNIP-5	290	30.62	9.15	0.37	6	W	V	A	R	F	S	A	Y	M	NPA	NPA	109	plas
SlNIP-6	345	37.22	8.30	0.44	6	W	I	A	R	F	S	A	Y	I	NPA	NPA	109	plas
SlNIP-7	295	30.63	9.06	0.50	6	S	I	A	R	F	T	A	Y	L	NPS	NPV	108	plas
SlNIP-8	260	27.82	8.91	0.68	6	W	V	A	R	F	S	A	F	V	NPA	NPA	112	vacu
SlNIP-9	306	31.66	8.34	0.46	6	T	I	A	R	L	T	A	Y	L	NPA	NPV	108	plas
SlNIP-10	231	25.45	9.62	0.45	4	/	S	G	R	F	S	A	Y	I	NPA	NPT	115	chlo
SlNIP-11	137	14.86	6.70	0.75	3	W	/	/	/	L	/	/	/	/	NPA	/	/	vacu
SlNIP-12	286	30.57	6.19	0.66	6	A	V	G	R	Y	S	A	Y	V	NPA	NPA	109	vacu
StLsi1	272	29.26	8.23	0.67	6	W	V	A	R	L	S	A	Y	I	NPA	NPA	109	plas
CsLsi1	288	30.55	9.40	0.33	6	G	S	G	R	L	T	A	Y	F	NPA	NPV	108	vacu
CsLsi6	261	27.62	6.05	0.45	6	C	S	G	R	M	S	A	Y	M	NPA	NPA	108	plas
HvLsi1	295	31.66	6.79	0.43	6	G	S	G	R	L	T	A	Y	F	NPA	NPA	108	plas
TaLsi1	295	31.67	6.79	0.42	6	G	S	G	R	L	T	A	Y	F	NPA	NPA	108	plas
SbLsi1	297	32.04	6.78	0.37	6	G	S	G	R	L	T	A	Y	F	NPA	NPA	108	plas
CmLsi1	297	32.04	6.78	0.37	6	G	S	G	R	L	T	A	Y	F	NPA	NPA	108	plas
ZmLsi1	295	31.76	6.79	0.35	6	G	S	G	R	L	T	A	Y	F	NPA	NPA	108	plas
OsLsi1	298	31.98	6.70	0.38	6	G	S	G	R	I	T	A	Y	F	NPA	NPA	108	plas
OsLsi6	298	31.83	7.00	0.49	6	G	S	G	R	L	T	A	Y	F	NPA	NPA	108	E.R
ZmLsi6	294	31.39	7.71	0.48	6	G	S	G	R	L	T	A	Y	F	NPA	NPA	108	plas
HvLsi6	300	32.25	7.75	0.44	6	G	S	G	R	L	T	A	Y	F	NPA	NPA	108	E.R

*Len* length, *MW* molecular weight, *pI* isoelectric point, *GR* grand average of hydropathicity, *TM* transmembrane domains, *ar/R* aromatic/arginine, *H2* helix2, *LE1* loop E1, *P1* position 1, *LB* loop B, *DB* distance, *LOC* localization.

For the members of aquaporin family, NPA domains will generate electrostatic repulsion to protons, and then form a channel, while ar/R selectivity filter and Froger’s positions will affect the specific substrate of the channel. All Si transporters identified in other species have two NPA domains, only with an exception for CsLsi1, which have NPV on Loop E with the alanine (A) in the third position replaced by threonine (T). However, NPA motif changed into NPV does not disturb Si transport activity of CsLsi1^35^. As for tomato, 11 of the 12 tomato NIPs all carried two NPA/S/V/T motifs, except for SlNIP-11 only having one NPA motif on Loop B. A specific length of 108 AA between the two NPA domains is a necessary and selectivity feature for Si among all Si-transporting plants^29^. These already validated Si-transporting Lsi1 proteins all presented 108 AA residues, such as CsLsi1, HvLsi1, TaLsi1, SbLsi1, CmLsi1, ZmLsi1 and OsLsi1. Whereas most of the tomato NIPs have 109 residues in the spacing, and only SlNIP-7 and SlNIP-9 have 108 AA. Moreover, SlNIP-8 has 112 AA and SlNIP-10 has 115 AA between the two NPA domains. However, the spacing itself is unlikely to be required for Si permeability. Supporting evidences showed that SlLsi1/SlNIP-1 possesses 109 AA in the spacing and shows transport activity for Si^30^.

For most members of NIP III subgroup from various species, including SlNIP-1 from tomato, four amino acids from helix 2 (H2), helix 5 (H5), and loop E (LE1 and LE2) that constitute ar/R selectivity filter are GSGR, except for CsLsi6, where a small G in the H2 is substituted with the bulkier C residue (Table 1). However, the residue at the H2 position is not critical for Si transport. For example, when glycine (G) was substituted by alanine (A) at H2 of OsLsi1, the transport activities for Si were unaffected^40^. Compared to residue at H2, residue at H5 is required for Si transport activity. When serine (S) at the H5 position was substituted by isoleucine (I), and the transport activity of OsLsi1 for Si was totally lost^40^. In addition, when both residues at the H2 and H5 were substituted, Si transport activities of OsLsi1 as well as transport activities for other solutes are completely lost. Most of tomato NIPs belong to NIP I subgroup possessing an ar/R filter consisting of W, V, A and R, except SlNIP-6 with ar/R selectivity filters of WIAR, and SlNIP-10 and SlNIP-11 whose ar/R selectivity filters are incomplete. For SlNIP-7, SlNIP-9 and SlNIP-12, which belong to the NIP II subgroup, their ar/R selectivity filters are SIAR, TIAR, and AVGR, respectively. It proposed us to speculate that SlNIP-6, SlNIP-7 and SlNIP-9 with I at H5, and SlNIP-10 and SlNIP-11with incomplete ar/R selectivity filter might have no transport activities for Si. It is worth testing the transport activities of SlNIP-1, SlNIP-2, SlNIP-3, SlNIP-4, SlNIP-5, SlNIP-8 with ar/R selectivity filter consisting of WVAR and SlNIP-12 with ar/R selectivity filter consisting of AVGR.

Froger’s positions are composed of five amino acids, which are located in highly conserved regions. Position 1, located in the terminal part of the third transmembrane segment, Positions 2 and 3 are located in loop E, and Positions 4 and 5 are located in the sixth transmembrane segment (^39^, Fig. 4). Residues in Position 3 (A) and Position 4 (Y) are conserved in almost all identified Si transporters. Position 2 is defined as a polar residue (S or T), which are usually the sites of phosphorylation of proteins. This residue may be responsible for the post-transcriptional modification and regulation of protein activity. However, the amino acid composition of Position 5 differs from SlNIP-1 to these already identified Si transporters. Position 5 is a nonaromatic residue in SlNIP-1, and this residue is aromatic in these already identified Si transporters (Table 1). Furthermore, within tomato NIPs, residues at Position 1 are also different between SlNIP-1 and members of tomato NIP I and NIP II subgroups. Residue at Postion 1 is an aromatic residue in NIP I and NIP II members, and this residue is not aromatic in SlNIP-1. Whether the variant residues at Position 1 and Position 5 in the Froger’s positions affect transport activities of NIPs or not, which need to be experimentally proved in further studies.

The predicted results of physical and chemical properties of these Si transporters as well as members of tomato NIP I and NIP II are recorded in Table 1. Except SlNIP-11 (137 AA and 14.86, respectively), the length of the encoded proteins mostly ranged from 231 to 345 amino acids, and their predicted molecular mass ranged from ~ 25.45 to 37.22 kDa. The isoelectric points of all 24 Si transporters ranged from 6.05 to 9.40, and the total average hydrophobicity of protein is between 0.33 and 0.75. The subcellular localization of these Si transporters and NIPs is predicted by WoLF online tool and recorded in Table 1. It is found that most of these proteins are located in plasma membrane, some are located in chloroplast membrane, vacuole membrane or endoplasmic reticulum. The difference between localizations may be related to the functional differences, and individual proteins may be involved in the transport of substances between plasma membrane and organelles.

### Tissue-specific expression analysis

**Figure 5:**
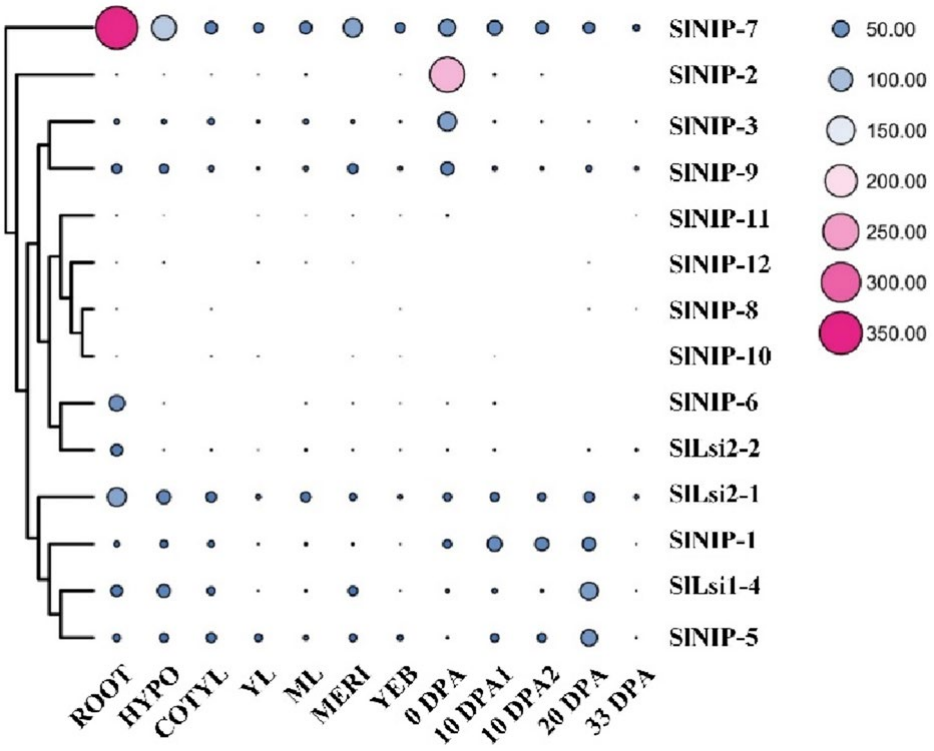
Expression profile of homologous genes of Si transporters in tomato in various organs available from public RNA-seq data. The expression data were log2 transformation of RPKM (reads per kilobase of exon model per million mapped reads) with TBtools v1.0986988, and a cluster dendrogram is shown on the left of each heat map. Different colors represent differences in gene expression. Red indicates high concentrations, whereas low relative concentrations are blue. *Root* whole root, *Hypo* hypocotyl, *Cotyl* Cotyledon, *YL* young leaf, *ML* mature leaf, *MERI* vegetative meristem, *YFB* young flower bud, *0 DPA* flower at anthesis, *10 DPA* fruit at 10 DPA, *20 DPA* fruit at 20 DPA, *33 DPA* fruit at 33 DPA.

To explore the functions of tomato NIP genes as well as the two SlLsi2 genes, we firstly investigated their expression atlas based on the RNA-seq data derived from the TOMATO FUNCTIOPNAL GENOMICS DATABASE in 11 various tissues of tomato including whole root, hypocotyl, cotyledons, young leaves, mature leaves, vegetative meristems, young flower buds, flowers at anthesis (0 DPA), 10 days post anthesis fruit (10 DPA), 20 days post anthesis fruit (20 DPA) and ripening fruit (33 DPA) (Fig. 5). *SlNIP-7* and *SlNIP-2* exhibited high and organ-specific expression pattern. *SlNIP-7* was highly expressed in all examined tissues, especially in root, indicating it may function in root. *SlNIP-2* was preferentially expressed highly in anthesis flowers. *SlNIP-5*, *SlNIP-9* and *SlLsi2-1* were constitutively expressed at a high level in nearly all tested tissues, and four genes (*SlNIP-8*, *SlNIP-10*, *SlNIP-11* and *SlNIP-12*) were expressed with very low transcript abundance. *SlNIP-3* was preferentially expressed in anthesis flowers, while *SlNIP-6* and *SlLsi2-2* was specially expressed in root. A similar expression pattern was observed on *SlNIP-4* and *SlNIP-5* genes, which showed relatively higher expression levels in root, hypocotyl, cotyledons, and fruit (20 DPA), but a lower level of expression in ripening fruit. *SlNIP-1* showed relatively higher expression levels in root, hypocotyl, cotyledons, anthesis flowers, immature fruit, but relatively lower expression levels in young leaves, mature leaves, vegetative meristems, young flower buds and ripening fruit, indicating SlNIP-1 may function during fruit development (Fig. 5).

**Figure 6:**
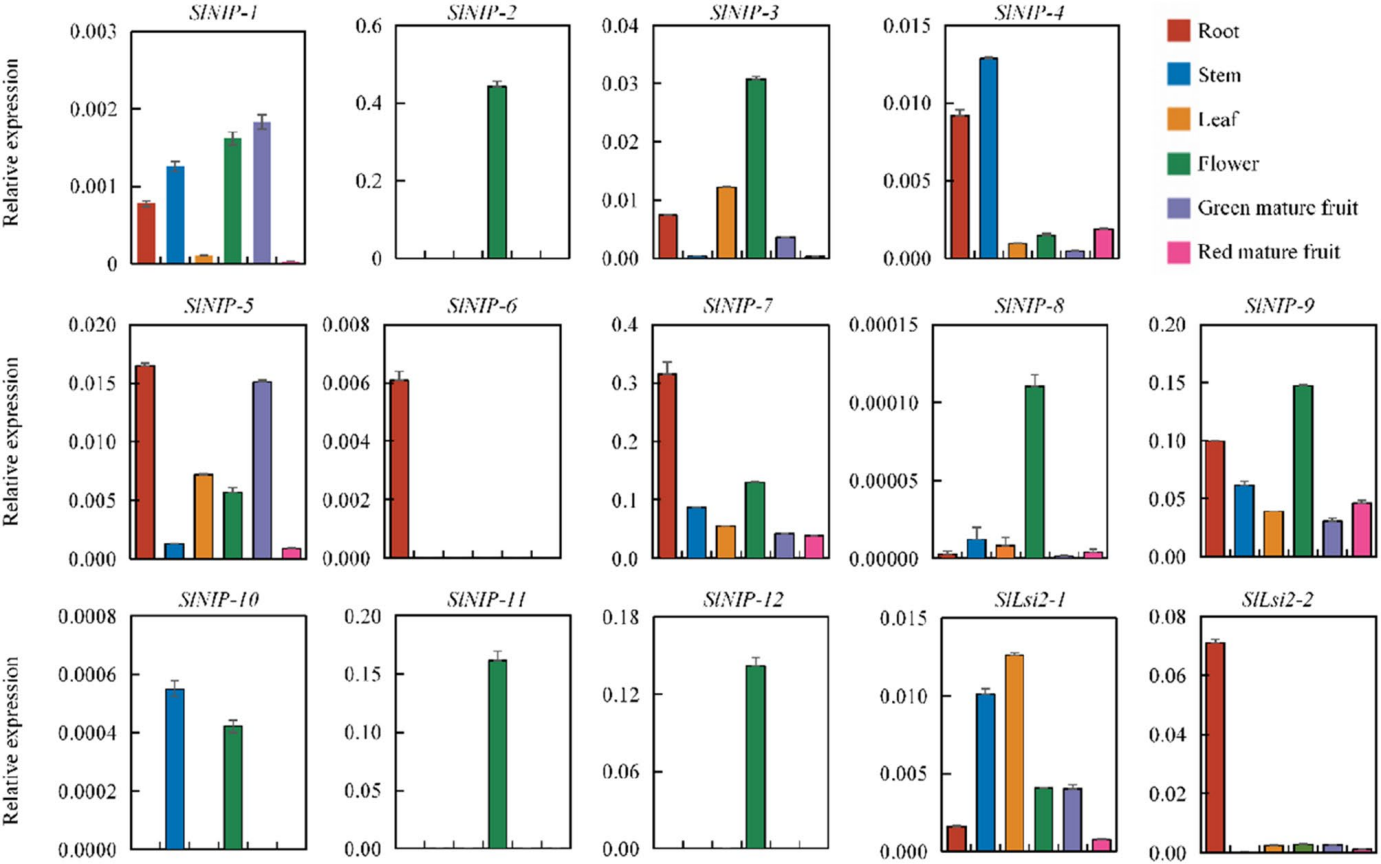
Expression patterns of homologous genes of Si transporters in tomato in different tissues were examined using qRT-PCR. Tomato *ACTIN2* gene was used as an internal standard. Values are means ± SD (n = 3). Gray represents the roots of tomato; yellow represents the stems of tomato; blue represents the leaves of tomato; red represents the flowers of tomato; green represents the green mature fruits of tomato; pink represents the red mature fruits of tomato.

To further explore the possible functions of tomato NIP genes, organ-specific expression profiles of these genes were further verified by RT-qPCR. We used 6 different tissues (root, stem, leaf, flower, mature green fruit, and red mature fruit). As shown in Fig. 6, expression patterns of most of these genes were consistent with the RNA-seq data in Fig. 5. Consistent with previous report, the already identified Si influx transporter *SlNIP-1* was mainly expressed in root relative to leaf, and Si efflux transporter *SlLsi2-2* was specific to root (Fig. 6^30^). Inconsistent with previous report^30^, another efflux transporter *SlLsi2-1* was expressed mainly in leaf but not in root, it may be due to *SlLsi2-1* was not a consecutive expression gene but an induced gene. Six genes (*SlNIP-2*, *SlNIP-3*, *SlNIP-8*, *SlNIP-9*, *SlNIP-11* and *SlNIP-12*) had similar expression patterns and demonstrated a high expression in flower. *SlNIP-4* was expressed strongly in root and stem, while *SlNIP-10* was expressed intensely in stem and flower. *SlNIP-5* was expressed significantly in root and green mature fruit, weakly in stem and red mature fruit, and moderately in leaf and flower, whereas, *SlLsi2-1* was expressed highly in stem and leaf, weakly in root and red mature fruit, and moderately in flower and green mature fruit. *SlNIP-6* like *SlLsi2-2* displayed root-specific expression patterns. *SlNIP-7* was constitutively expressed at a high level in almost all tested tissues, and showed relatively higher expression levels in root. Above all, different members of the tomato NIP genes showed distinct expression patterns, indicating these genes may function in many aspects of tomato growth and development.

### Promoter analysis

**Figure 7:**
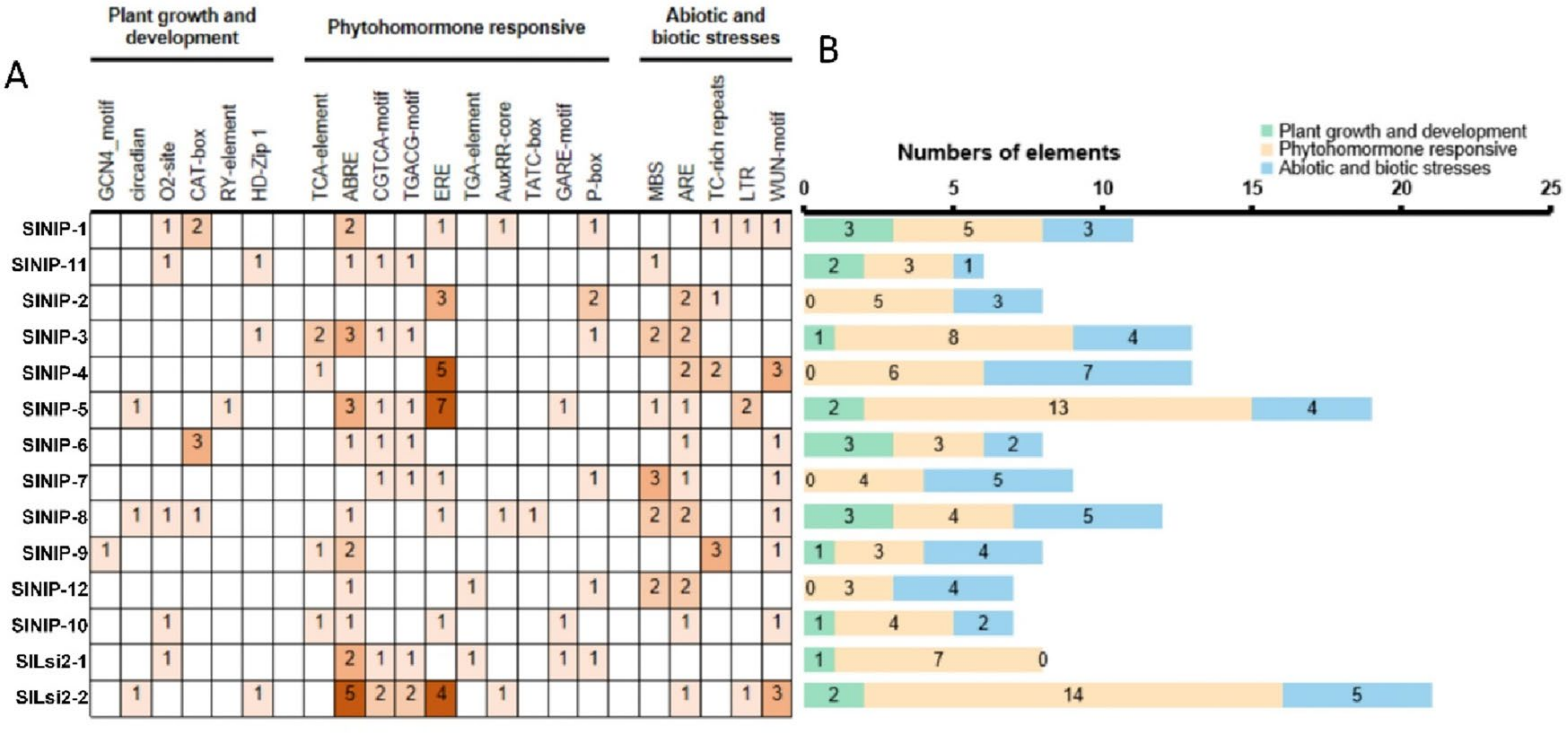
Prediction of cis-acting elements in promoter regions of Si transporter homologues in tomato. (**A**) The different colors and numbers of the grid indicated the numbers of rech predicted elements in these Si transporter homologues promoter region. (**B**) The different colored histogram represented the sum of the cis-acting elements in each category.

Specific cis-acting elements can combine with particular transcription factors to regulate downstream gene transcription and expression. In order to determine the promoter cis-acting elements of 14 tomato Si transporter homologous proteins, the 2000 bp sequences upstream of the start site (ATG) of these genes were downloaded as putative promoters from SGN database. The promoter sequences were analyzed by using PlantCARE online tool, and the identified cis-acting elements were sorted into three groups related to plant growth and development, hormone response and biotic/abiotic stresses (Fig. 7). Cis-acting elements related to plant growth and development included endosperm expression element (GCN4_motif), circadian rhythm regulation element (circadian), zein metabolism regulation element (O_2_-site), meristem expression element (CAT-box), seed-specific regulation element (RY-element) and mesophyll cell differentiation element (HD-Zip1). There are only 0–3 cis-acting elements of this group in promoters of all 14 tomato Si transporter homologous genes, which indicates that these genes may be less induced by signaling from the specific growth and development stage of plants. Cis-acting elements belonging to hormone response included salicylic acid responsive element (TCA-element), abscisic acid (ABA) responsive element, methyl jasmonate responsive element (CGTCA-motif, TGACG-motif), ethylene responsive element (ERE), auxin responsive element (TGA-element, AuxRR-core) and gibberellin responsive element (TATC-box, GARE-motif, P-Box), which has 3–14 cis-acting elements of this group in the promoters of 14 tomato Si transporter homologues, indicating that these genes are possibly sensitive to plant hormones. Eleven tomato Si transporter homologues except *SlNIP-2*, *SlNIP-4* and *SlNIP-7* all have ABA responsive elements, while eight tomato Si transporter homologues except *SlNIP-3*, *SlNIP-6*, *SlNIP-9*, *SlNIP-11*, *SlNIP-12* and *SlLsi2-1* all have ethylene responsive element (ERE). ABA and ethylene play vital roles in plant growth and development as well as in mediating plant response to a wide range of stresses. It should be noted that tomato Si transporter homologues may be regulated by ABA or ethylene and participate ABA or ethylene-mediated plant development and stress responsiveness. Cis-acting elements related to biotic/abiotic stress included drought response element (MBS), anaerobic induction response element (ARE), defense and stress response element (TC-rich repeats), low temperature response element (LTR) and mechanical injury response element (WUN-motif). There are 1–7 cis-acting elements of this group in the promoters of 14 tomato Si transporter homologues. Among them, three WUN-motifs, MBS, TC-rich repeats were found in *SlNIP-4*, *SlLsi2-2*, *SlNIP-7* and *SlNIP-9* promoters, respectively. The cis-regulatory elements identified in the promoters of tomato Si transporter homologues suggested that they may play an important role in hormone signaling and various stress response.

### Subcellular localization of SlNIP-1 and its mutated proteins

**Figure 8:**
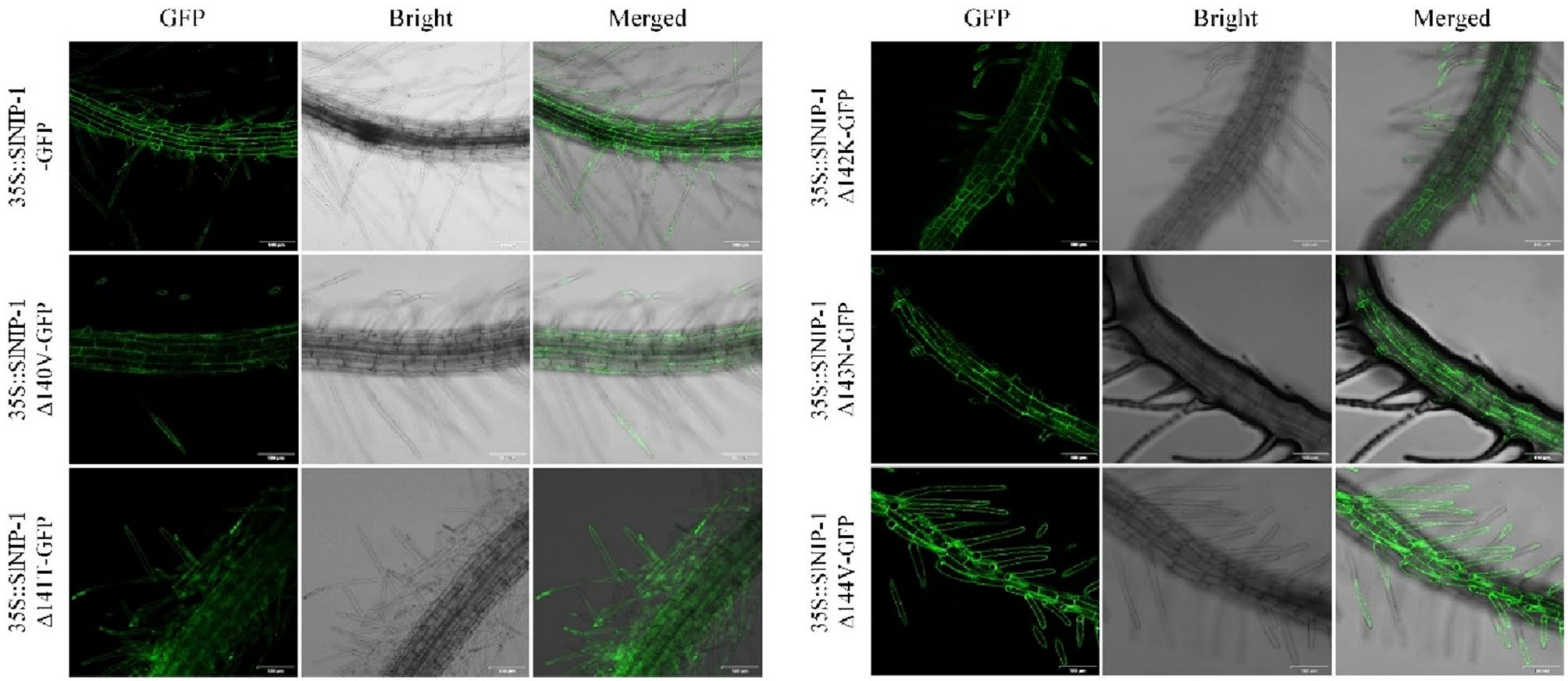
Subcellular localization of SlNIP-1 protein and its mutations of site-specific deletion. The roots of seven-day-old seedlings were used and the fluorescence were monitored. Bars 100 µm. Vectors of *35::SlNIP-1-GFP*, *35::SlNIP-1Δ140V-GFP*, *35::SlNIP-1Δ141T-GFP*, *35::SlNIP-1Δ142K-GFP*, *35::SlNIP-1Δ143N-GFP*, and *35::SlNIP-1Δ144V-GFP* were introduced into Arabidopsis (*Arabidopsis thaliana*) ecotype Columbia mediated by *Agrobacterium tumefaciens strain* GV3101. T_1_ transgenic plants were selected by genotyping.

Plasma membrane location is required for the uptake of Si by Lsi1 transporters. Functional Lsi1 Si influx transporters are unexceptionally localized in the plasma membrane, such as OsLsi1^13^, HvLsi1^33^, ZmLsi1^34^, TaLsi1^22^, SlLsi1^29^, CsLsi1^35^, and NsLsi1^41^. Subcellular localization can largely affect the Si transport activities. CmLsi1 from the bloomless rootstock that was localized at the plasma membrane have transport activity, whereas the one from the bloomless rootstock was localized at the endoplasmic reticulum and had no transport activity for Si. In order to verify the subcellular localization of tomato SlNIP-1, we cloned the 849 bp CDS sequence of *SlNIP-1*, and introduced it into the vector *pGWB5* to obtain the GFP fusion vector *SlNIP-1-pGWB5* (*35S::SlNIP-1-GFP*). The vector was transformed into *Agrobacterium tumefaciens* GV3101 to generate transgenic *Arabidopsis* plants by floral dip method. T_1_ transgenic *Arabidopsis* plants were used for observing GFP fluorescence signal. Consistent with WoLF's prediction in Table 1 and previous report^29^, SlNIP-1 protein was localized in plasma membrane (Fig. 8). A 108-AA spacing between NPA domains is essential to Si influx transport activity for SlLsi1^29^. Predicted by PROVEAN, we found five site deletions (140 V, 141 T, 142 K, 143 N and 144 V) result in neutral effects. Then, the localization of variant SlNIP-1 mutants of single amino acid deletion was confirmed by transgenic plants. As the results showed that, like SlNIP-1, these five mutated SlNIP-1 proteins (SlNIP-1Δ140V, SlNIP-1Δ141T, SlNIP-1Δ142K, SlNIP-1Δ143N, and SlNIP-1Δ144V) with a spacing of 108 AA were also located on the plasma membrane (Fig. 8). In 2015, Deshmukh et al. had reported that deletion 140 V in SlNIP-1 exhibited Si permeability relative to the native SlNIP-1 protein^29^. Thus, the function of the other four SlNIP-1 mutants need to be tested.

## Discussion

Tomato Si transporter homologues SlNIP-1 to SlNIP-12 belong to NIP subfamily (Figs. 1, 3). Six of the twelve proteins with ar/R filter consisting of Trp (H2), Val (H5), Ala (LE1), and Arg (LE2), WVAR, as well as SlNIP-6 with WIAR, belong to the NIP I subgroup, whereas SlNIP-7 with SIAR, SlNIP-9 with TIAR, and SlNIP-12 with AVGR were classified into NIP II group^42,43^. Additionally, SlNIP-1 with GSGR belong to NIP III group^28^. Compared to NIP III subgroups, which have small amino acid residues Gly in H2 and Ser in H5; members of NIP I subgroups have two bulky residues Trp and Val in H2 and H5 positions, respectively^23^. So, considering the composition of ar/R filter, we propose that most members of tomato NIPs possessing WVAR (WIAR in SlNIP-6) do not have transport activities for silicic acid, but may transport small molecules of water, glycerol and lactic acid as members in NIP I. To support the propose, one study showed that replacement of AIGR by WIGR endows AtNIP6;1 with water transport activity, suggesting the possible water-transport activity as well as glycerol transport of SlNIP-6 which was presented by WIAR of the ar/R region.

The Arabidopsis NIP II subgroup has three member genes: AtNIP5;1, AtNIP6;1, and AtNIP7;1^23^, and each of them has an ortholog in tomato, represented by SlNIP-7, SlNIP-9 and SlNIP-12, respectively^43^. Current study further confirmed the remarkable orthologous relationships between AtNIP5;1, AtNIP6;1, AtNIP7;1 and SlNIP-7, SlNIP-9, SlNIP-12. Firstly, structural similarity is within pore determinant regions, such as SlNIP-7 and AtNIP5;1 share the a/R filter of SIAR; SlNIP-9 and AtNIP6;1 share the a/R filter of TIAR; SlNIP-12 together with AtNIP7;1 share the a/R filter of AVGR (Table 1). Secondly, similar tissue expression profiles, RT-qPCR combined with RNA-seq data showed that SlNIP-7 was mainly expressed in the roots, SlNIP-9 was constitutively expressed in all examined tissues with high expression level in stem, and SlNIP-12 was specifically expressed in flower (Fig. 5), which were similar to previous studies^43–45^. Thirdly, similar to previous observations with AtNIP5;1^45^, AtNIP6;1^44^ and AtNIP7;1^43^, SlNIP-7 and SlNIP-9 were predicted to be localized on plasma membrane, and SlNIP-9 was predicted to be localized on vacuole membrane (Table 1). As AtNIP5;1, AtNIP6;1, and AtNIP7;1 were reported to mediate boron uptake and contribute to the development of root, rosette leaves, inflorescences and pollen grain^27,43,44^. In summary, the results suggested SlNIP-7, SlNIP-9 and SlNIP-12 might act as major channel proteins mediating boric acid transport. It will be important to investigate the substrate specificity of the SlNIP-7, SlNIP-9 and SlNIP-12 and the possible involvement of SlNIP-7, SlNIP-9 and SlNIP-12 in plant growth and development.

In addition to ar/R selectivity filter, NPA motif and inter‐NPA distance can also affect the Si permeability of Lsi1 channels. It has been reported that two NPA motifs localized at loop B and loop E form part surface of the narrow aqueous pore^46^. Residues composed of NPA motifs influence the size of constriction filter, and then the specificity of substrates. For example, the pore diameter of the NPA regions of AtNIP6;1 is narrower than that of Nodulin 26, which may result from a larger Val substitution for the small Ala in the second NPA motif (loop E) of AtNIP6;1 compared with Nodulin 26^26^. Most members of the tomato NIPs have two conserved NPA motifs, while the first NPA motif in SlNIP-4 and SlNIP-7 was changed to NPS. In addition, the second NPA motif in SlNIP-7 was also replaced by NPV. Moreover, the second motif in SlNIP-9 and SlNIP-10 was changed to NPV and NPT, respectively. However, it seems that the second NPA motif is not a crucial factor for the substrate specificity of NIP proteins. In the case of AtNIP6;1, a substitution of Ala for Val in the second NPA motif has little effect on the transport selectivity of AtNIP6;1^26^.

An inter‐NPA distance of 108-AA is a common feature that is critical for the function of Si transporters. SlNIP-1/SlLsi1 owned an ar/R filter composing of GSGR belonging to the NIP III group and might be a Si transporter, but 109 AA in the inter‐NPA region of SlNIP-1 may cause the loss function of Si transport activities. However, Si permeability of SlLsi1 is still controversial^29,30^. In addition to the precise space between the NPA domains, other molecular determinants can affect the function of Lsi1 transporters. NsLsi1 from tobacco possessing typical molecular signatures, a GSGR selectivity filter and a 108 AA inter‐NPA space, were found to be Si‐impermeable^41^. Further investigation showed that Si transport activities of NsLsi1 can be compromised by P125F substitution. P125F substitution increased plasma membrane localization of NsLsi1^P125F^ compared to NsLsi1^WT^, thus enhanced the transport capacity^41^. It was also reported that analysis of boric acid permeability of AtNIP7;1 by expressing AtNIP7;1 in *Xenopus laevis* oocytes and proteoliposomes yielded contrasting results, explaining by some factors influencing the pore structures of the AtNIP7;1 changed in this two systems^43^. As for the case of SlNIP-1, other than the molecular characteristics of protein sequences, determining factors, such as biochemical properties as well as posttranslational modification, which affect the function of SlNIP-1 may be changed due to different analysis systems and methods. Besides, deletion 140 V in SlLsi1/SlNIP-1 restored its Si permeability^29^. In the future, the activity of SlNIP-1Δ141T, SlNIP-1Δ142K, SlNIP-1Δ143N, and SlNIP-1Δ144V are worth testing. Finally, definition of the in vivo transport properties of SlNIP-1 will require further detailed investigation.

Tissue-specific expression pattern reflects the function of genes to some extent. We conducted RNA-seq and RT-qPCR analysis, discovering some interesting trends and expression patterns of 14 tomato Si transporter homologues (Figs. 5, 6). SlNIP-1 and SlLsi2-2 were mainly expressed in root relative to leaf, which is consitent with their function reported in previous study^30^. SlLsi2-1 was found mainly expressed in leaf but not root, which did not match previous results^30^. This discrepancy may reflect that SlLsi2-1 was not expressed constantly but can be induced by some stimulation. The high expression pattern in root was also found in SlNIP-4, SlNIP-5, and SlNIP-6, implying their roles in root. Indeed, AtNIP2;1 from *Arabidopsis* was a root-specific expression gene, and it was proved to be a lactic acid efflux channel and is necessary for an optimum response to low oxygen stress^47^. Thus, considering SlNIP-4, SlNIP-5 and SlNIP-6 both belong to the NIP I family and with similar root-specific expression as AtNIP2;1, we propose that SlNIP-4, SlNIP-5 and SlNIP-6 may be involved in abiotic stress response in root.

SlNIP-2, SlNIP-8, and SlNIP-11 exhibited an almost undetectable low expression level in most examined tissues but show high expression levels in flower, suggesting these genes may play specific roles in flower. Similarly, SlNIP-3 and SlNIP-10 were also mainly expressed in flower. Polygenetic tree indicated that SlNIP-2, SlNIP-3 and SlNIP-11 were highly homologous with AtNIP4;1 and AtNIP4;2. It has been reported that AtNIP4;1 and AtNIP4;2 were expressed in flower specific to pollen grains and pollen tubes, and characterized as important regulators for the proper pollen development, pollen germination, and pollen tube growth^48^. Similar to AtNIP4;1 and AtNIP4;2, SlNIP-2, SlNIP-3 and SlNIP-11 may participate in the male gametophyte development.

## Conclusions

There are 43 aquaporins including 8 NIP I proteins, 3 NIP II proteins, and 1 NIP III protein in tomato. SlNIP-1 was the reported Si influx transporters SlLsi1. Mutated SlNIP-1 proteins of single amino acid deletion—SlNIP-1Δ141T, SlNIP-1Δ142K, SlNIP-1Δ143N, and SlNIP-1Δ144V—resembled the native SlNIP-1 showing plasma membrane localization. Though polygenetic tree analysis, conserved structure analysis, and gene expression patterns analysis, we predict SlNIP-7, SlNIP-9, SlNIP-12 may be boric acid facilitators, and SlNIP-2, SlNIP-3 and SlNIP-11 may be involved in the pollen development. Considering the important biological functions of NIP subfamily of plant aquaporins, further analysis of the functions of these genes in specific tissue is deserved. In addition, we also propose that functional identification of SlNIP-1, SlLsi2-1 and SlLsi2-2 should be obtained through transgenic plants and furthermore experimental studies.

## Materials and methods

### Screening and identification of Lsi1 and Lsi6 homologues and Lsi2 homologues in tomato

The amino acid sequences of Si transporters Lsi1, Lsi2 and Lsi6 of rice and cucumber were obtained from NCBI database (https://www.ncbi.nlm.nih.gov/). Sequences of tomato were searched by the BLAST program provided by tomato genome database SGN (https://solgenomics.net/), and then the sequences of retrieved homologous proteins with E value less than e^−6^ was selected. The amino acid sequences of barley (*Hordeum vulgare* L.), wheat (*Triticum aestivum* L.), corn (*Zea mays* L.), sorghum (*Sorghum bicolor* L.), pumpkin (*Cucurbita moschata* L.) and potato (*Solanum tuberosum* L.) were downloaded from NCBI database.

### Phylogenetic tree construction and chromosome location

Sequence alignments of Si transporters of tomato and other species were carried out by ClustalX2 software (http://www.clustal.org/)^49^, and sequence alignment and evolutionary tree construction were further carried out by MEGA7.0 software (https://www.megasoftware.net/)^50^. Neighbor-joining method was selected as the evolutionary tree construction method with 1000 bootstrap replications. Finally, the phylogenetic tree was illustrated using Interactive Tree of Life (IToL, http://itol.embl.de) and Notepad++ v7.8.8 software (https://notepad-plus-plus.org/downloads/v7.8.8/). The chromosomal locations of tomato Lsi1, Lsi2 and Lsi6 homologous genes were searched in tomato genome database SGN, and the distribution of gene location on chromosome was drawn by MapInspect 1.0 software (https://mapinspect.software.informer.com/).

### Analysis of exon–intron structure and conserved motifs

The gene sequences of tomato Si transporter homologous proteins were searched from tomato genome database SGN, and the exon and intron of these genes were found on GSDS website (http://gsds.cbi.pku.edu.cn/); the motifs of these proteins was searched by MEME website (https://meme-suite.org/meme/). The method was set to Any Number of Repetitions, and the number of motifs searched was set to 10. The transmembrane domains were analyzed by TMHMM online tool (http://www.cbs.dtu.dk/services/TMHMM/) prediction; subcellular localizations were predicted by WoLF PSORT Tool (https://wolfpsort.hgc.jp/). The basic physical and chemical properties of protein were analyzed by ProtParam online tool (http://web.expasy.org/protparam/); the positions of ar/R selectivity filter and Froger’s residues were marked after sequence alignments.

### Evolutionary analysis by maximum likelihood method

The evolutionary history was inferred by using the Maximum Likelihood method and JTT matrix-based model. The tree with the highest log likelihood (− 14,866.98) is shown. The percentage of trees in which the associated taxa clustered together is shown next to the branches. Initial tree(s) for the heuristic search were obtained automatically by applying Neighbor-Join and BioNJ algorithms to a matrix of pairwise distances estimated using the JTT model, and then selecting the topology with superior log likelihood value. This analysis involved 33 amino acid sequences. There were a total of 474 positions in the final dataset. Evolutionary analyses were conducted in MEGA 7.0.

### Tissue-specific expression analysis

The expression patterns analysis of tomato Si transporter homologue genes in different tissues of different development stages of tomato was carried out according to RNA-seq data from Tomato Functional Genomics Database (TFGD, http://ted.bti.cornell.edu/cgi-bin/TFGD/digital/home.cgi). Transcriptome analysis of 11 tissues in wild species *S. pimpinellifolium*, LA1589 using Illumina RNA-seq. The tissues include: newly developed leaves around 5 mm long, mature green leaflets, flower buds 10 days before anthesis or younger, flowers at anthesis, fruit of 10 days post anthesis (DPA), 20 DPA fruit, and breaker stage ripening fruit. RPKM values are an average of 4 replicates. Gene expression data of RPKM values were log2 transformed before analysis, and heatmaps were generated by TBtools v1.0986988 software^51^.

### Plant materials and growth conditions

Tomato (*Solanum lycopersicum*) cv. Ailsa Craig (AC) was used as the wild type in this study. Seeds of tomato cultivar AC (Accession LA2838A) were obtained from the Tomato Genetics Resources Center at the University of California, Davis (https://tgrc.ucdavis.edu/). Tomato seeds were placed on moistened filter paper for 48 h for germination. Tomato seedlings were transferred to growth chambers and maintained under a long-day photoperiod (16 h of light/8 h of dark) with a white light intensity of 200 mmol photons m^−2^ s^−1^ at 25 °C during the subjective day and at 18 °C during the subjective night.

Arabidopsis (*Arabidopsis thaliana*) ecotype Columbia was used as the wild type in this study. *Arabidopsis* plants were grown in Murashige and Skoog (MS) media at 22 °C under a long-day (16 h light/8 h dark) photoperiod (light intensity of 120 mM photons m^−2^ s^−1^).

The plant materials were well used for research project and comply with relevant institutional, national, and international guidelines and legislation.

### RNA extraction and quantitative RT-PCR (qRT-PCR) analysis

TRIzol reagent (Invitrogen) was used for RNA extraction. For quantitative real-time polymerase chain reaction (qRT-PCR) analysis, one microgram of DNA-free RNA was transcribed into first strand cDNA by Prime-Script RT Master Mix (TaKaRa). The qRT-PCR was carried out with the UVP ChemStudio (analyticjena) using TB Green Premix Ex Taq (TaKaRa). The reaction conditions were 95 °C for 30 s, and 40 cycles at 95 °C for 5 s, 60 °C for 30 s. Expression levels of target genes were normalized relative to *ACTIN2* gene. Primers used to quantify gene expression levels are listed in Supplementary Table S4. Each reaction was performed with three biological replicates.

### Cis-elements analysis

Regions 2000 bp upstream of the start codon (ATG) of each gene were downloaded in the tomato genome database SGN as the predicted promoter sequences. Cis-acting elements of the promoters were predicted by PlantCARE (http://bioinformatics.psb.ugent.be/webtools/plantcare/html/).

### Plasmid construction and plant transformation

To generate the *35S::SlNIP-1-GFP* construct, the coding sequence without termination codon of *SlNIP-1* was cloned into pENTR and recombined with the binary vector *PGWB5* (35S promoter, C-GFP). A similar approach was used to generate the *35S::SlNIP-1Δ140V-GFP* (35S promoter, C-GFP), 3*5S::SlNIP-1Δ141T-GFP*, *35S::SlNIP-1Δ142K-GFP*, *35S::SlNIP-1Δ143N-GFP*, *35S::SlNIP-1Δ144V-GFP* constructs. All primers used for DNA construct generation are listed in Supplemental Table S4. The above constructs were then transformed into *Agrobacterium tumefaciens* strain GV3101, which was used for transformation of *Arabidopsis* plants via the floral dip method. T_1_ transgenic plants were selected based on their resistance to hygromycin.

### Microscopy observations

For subcellular localization analysis, roots of 10-day-old T_1_ transgenic plants with corresponding constructs were observed and imaged under a laser-scanning confocal microscope (Olympus fluoview FV3000). For imaging GFP, the 488-nm lines of the laser were used for excitation, and emission was detected at 510 nm.

## Supplementary Information

Supplementary Information.

## Data Availability

The amino acid sequences analyzed in the current study are available in the Sol Genomics Network repository (https://solgenomics.net/), the NCBI database (https://www.ncbi.nlm.nih.gov/), the Arabidopsis Information Resource (TAIR) (http://www.arabidopsis.org/) and the rice genome annotation database (http://rice.uga.edu/), respectively. The accession numbers are included in the Supplementary Tables.
