# Supplementary material for: The structure, function and expression analysis of the nodulin 26-like intrinsic protein subfamily of plant aquaporins in tomato
Source: Sci Rep. 2022 Jun 2;12:9180. doi: 10.1038/s41598-022-13195-0 (PMC9163140; doi:10.1038/s41598-022-13195-0)
Supplement: Supplementary file 1 — Supplementary Information. [file 41598_2022_13195_MOESM1_ESM.pdf]

The structure, function and expression analysis of the nodulin 26-like intrinsic protein subfamily of plant aquaporins in tomato

Yuxiang Zhang, Shihong Fei, Yunmin Xu, Yong He, Zhujun Zhu* and Yuanyuan Liu*
Collaborative Innovation Center for Efficient and Green Production of Agriculture in Mountainous Areas of Zhejiang Province, College of Horticulture Science, Zhejiang A&F University, Hangzhou 311300, Zhejiang, China

***** Correspondence: yyliu@zafu.edu.cn, zhuzj@zafu.edu.cn.

Supplementary tables and figures legends

Supplementary Table S1. The accession numbers of already reported 17 Lsi1/Lsi2/Lsi6 Si transporters identified in rice, barley, wheat, maize, sorghum, pumpkin, potato and cucumber from NCBI database.

Supplementary Table S2: Nomenclature of tomato homologous genes of Si transporter in Clade II and Clade III, and their accession numbers in the Sol Genomics Network.

Supplementary Table S3. The accession numbers of NIPs in Arabidopsis and rice that were used for constructing evolutionary analysis of NIP subfamily from tomato, Arabidopsis and rice.

Supplementary Table S4. Primers used in this study.

Supplementary Fig. S1: Characterization of the 45 homologous genes of Si transporters in tomato.

Supplementary Table S1. The accession numbers of already reported 17 Lsi1/Lsi2/Lsi6 Si transporters identified in rice, barley, wheat, maize, sorghum, pumpkin, potato and cucumber from NCBI database.

Locus	Name
XP_015626173.1	*OsLsi1*
XP_044954480.1	*HvLsi1*
XP_037452918.1	*TaLsi1*
NP_001105637.1	*ZmLsi1*
XP_002454286.1	*SbLsi1*
XP_022934019.1	*CmLsi1*
XP_006344325.1	*StLsi1*
NP_001267699.1	*CsLsi1*
XP_015644134.1	*OsLsi6*
XP_044961713.1	*HvLsi6*
NP_001105020.1	*ZmLsi6*
APT69294.1	*CsLsi6*
XP_015632217.1	*OsLsi2*
XP_044983251.1	*HvLsi2*
NP_001183945.1	*ZmLsi2*
XP_022944823.1	*CmLsi2*
XP_004140769.1	*CsLsi2*

Supplementary Table S2. Nomenclature of tomato homologous genes of Si transporter in Clade II and Clade III, and their accession numbers in the Sol Genomics Network.

Locus	Name
Solyc03g013340	*SlNIP-1*
Solyc02g091420	*SlNIP-2*
Solyc05g008080	*SlNIP-3*
Solyc03g005980	*SlNIP-4*
Solyc02g071920	*SlNIP-5*
Solyc06g073590	*SlNIP-6*
Solyc08g013730	*SlNIP-7*
Solyc12g057050	*SlNIP-8*
Solyc03g117050	*SlNIP-9*
Solyc02g071910	*SlNIP-10*
Solyc02g063310	*SlNIP-11*
Solyc01g079890	*SlNIP-12*
Solyc06g036100	*SlLsi2-1*
Solyc03g025350	*SlLsi2-2*

Supplementary Table S3. The accession numbers of NIPs in Arabidopsis and rice that were used for constructing evolutionary analysis of NIP subfamily from tomato, Arabidopsis and rice.

Locus	Name
AT1G31885.1	*AtNIP3;1*
AT1G80760.1	*AtNIP6;1*
AT2G34390.1	*AtNIP2;1*
AT3G06100.1	*AtNIP7;1*
AT4G10380.1	*AtNIP5;1*
AT4G18910.1	*AtNIP1;2*
AT4G19030.1	*AtNIP1;1*
AT5G37810.1	*AtNIP4;1*
AT5G37820.1	*AtNIP4;2*
Os02g0232900	*OsNIP1;1*
Os01g0202800	*OsNIP1;2*
Os05g0205050	*OsNIP1;3*
Os06g0552700	*OsNIP1;4*
Os02g0745100	*OsNIP2;1*
Os06g0228200	*OsNIP2;2*
Os10g0513200	*OsNIP3;1*
Os08g0152000	*OsNIP3;2*
Os08g0152100	*OsNIP3;3*
Os12g0204100	*OsNIP3;4*
Os08g0152100	*OsNIP3;5*
Os01g0112400	*OsNIP4;1*

Supplementary Table S4. Primers used in this study.

Primer name	Primer sequence (5’-3’)
ACTIN2-qPCR-F	TTGCTGACCGTATGAGCAAG
ACTIN2-qPCR-R	GGACAATGGATGGACCAGAC
SlNIP-1-qPCR-F	TCTGCTGTCGCCACTGATAC
SlNIP-1-qPCR-R	TACTGGCCCCGCCAAAATAG
SlNIP-2-qPCR-F	GGATTTCTGTGACGTGGGGA
SlNIP-2-qPCR-R	GCGGGGTTAAAATGTGCTCC
SlNIP-3-qPCR-F	TAGCAGAGGCGATAGGAACG
SlNIP-3-qPCR-R	CCCCATGCCACACATATTCC
SlNIP-4-qPCR-F	AGCCATAACTTGGGGCCTTG
SlNIP-4-qPCR-R	GGGAACCTTTTGACAGAGGCA
SlNIP-5-qPCR-F	GCAGTTTTGTCACGGTTCCC
SlNIP-5-qPCR-R	CTTTATCGGCATTCACCGCC
SlNIP-6-qPCR-F	AGCAGAGTTTGTGGGAACGTA
SlNIP-6-qPCR-R	AAACAGACGCGATTCCAGGT
SlNIP-7-qPCR-F	GGGAGAGTTGGCTGGCATAG
SlNIP-7-qPCR-R	GCCCCAAAGTTCGTACTGGA
SlNIP-8-qPCR-F	TGACGATCGAGCGACCAAAG
SlNIP-8-qPCR-R	AGCTCCAGTAATTGGCCCTG
SlNIP-9-qPCR-F	GGTGCTCCTCTCTTTGGTGG
SlNIP-9-qPCR-R	CGGTTCAACACCGAAGCATTT
SlNIP-10-qPCR-F	CAAGTCGTGGGAGCAACTCT
SlNIP-10-qPCR-R	GTTTATCCATTGCGGTGCCA
SlNIP-11-qPCR-F	TTTCACCGTCTTTCGTCGCT
SlNIP-11-qPCR-R	TGCTAAAGTAACGCCTGCCA
SlNIP-12-qPCR-F	TTGGCAGAAGCATTAGGGACA
SlNIP-12-qPCR-R	AGTGCTGCTGTGGTTGCATA
SlLsi2-1-qPCR-F	TGGTCCTGTCAAATGTGGCT
SlLsi2-1-qPCR-R	CCACGCTTTCTGCTCACTCT
SlLsi2-2-qPCR-F	CCCACCTCATCCGTTCCTTT
SlLsi2-2-qPCR-R	CTCCCGAACGTGATCTTGCT
SlNIP-1-F	ATGGAGAGTGAAGGAGGGAAC
SlNIP-1-R	GAGGGTAACACATTGTTCTTCATCG
SlNIP-1-Δ140V-F	TCATCCAACTAAAAATGTGGGAACT
SlNIP-1-Δ140V-R	AGTTCCCACATTTTTAGTTGGATGA
SlNIP-1-Δ141T-F	TCATCCAGTAAAAAATGTGGGAACTAC
SlNIP-1-Δ141T-R	AGTTCCCACATTTTTTACTGGATGAA
SlNIP-1-Δ142K-F	TCATCCAGTAACTAATGTGGGAACTAC
SlNIP-1-Δ142K-R	AGTTCCCACATTAGTTACTGGATGAA
SlNIP-1-Δ143N-F	TCATCCAGTAACTAAAGTGGGAACTAC
SlNIP-1-Δ143N-R	AGTTCCCACTTTAGTTACTGGATGAA
SlNIP-1-Δ144V-F	TCATCCAGTAACTAAAAATGGAACTAC
SlNIP-1-Δ144V-R	AGTTCCATTTTTAGTTACTGGATGAA

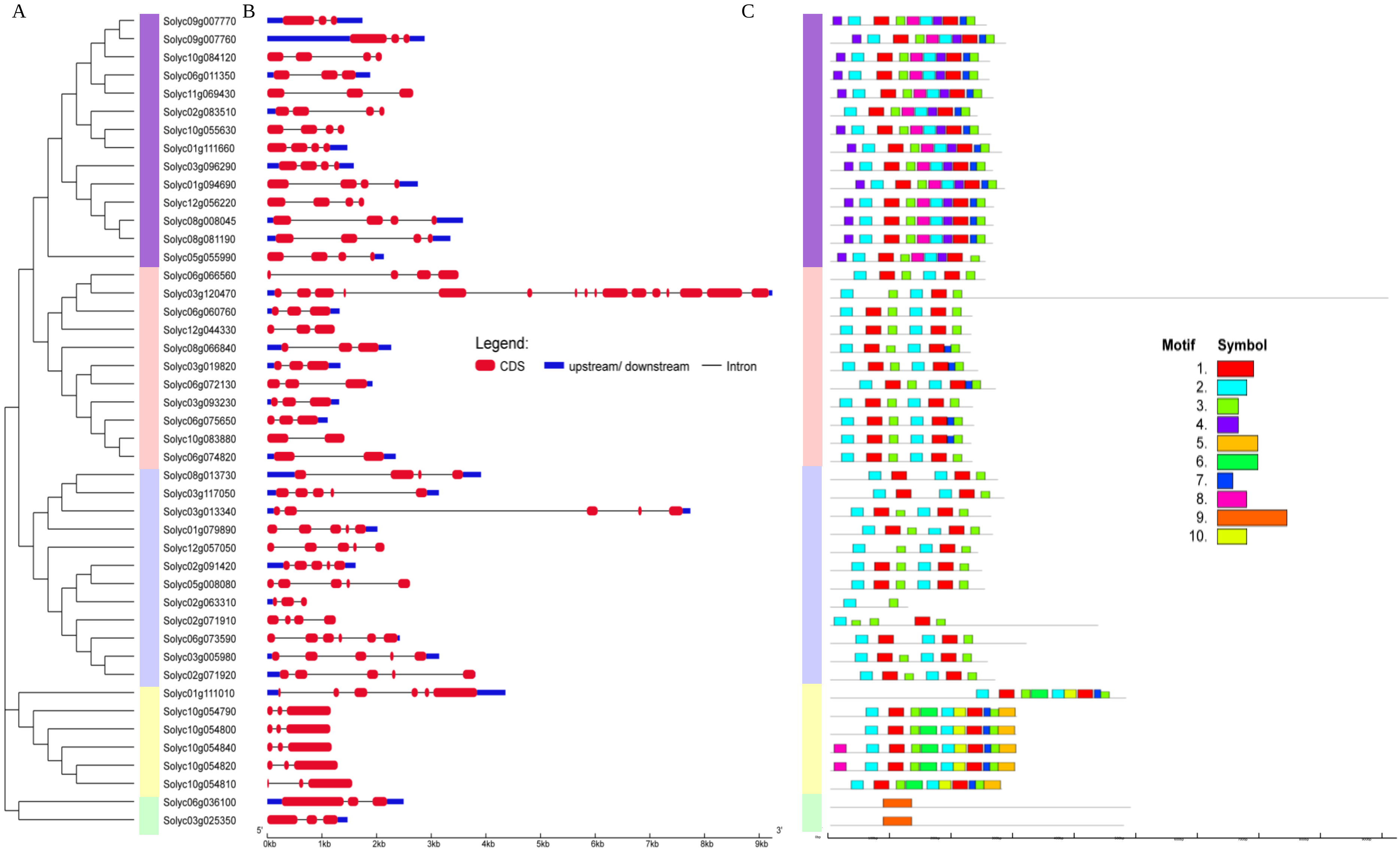

Supplementary Figure S1. Characterization of the 45 homologous genes of Si transporters in tomato. (A) Phylogenetic analysis of 45 Si transporter homologous proteins in tomato. The phylogenetic tree was constructed based on the full-length protein sequences of silicon transporter homologous proteins in tomato using MEGA 5 software. Each of clades is shown in a specific color bar. Yellow represents Clade I, green represents Clade II, blue represents Clade III, red represents Clade IV, and purple represents Clade V. (B) Gene structure of Si transporter homologous proteins in tomato. b The organization of the Exon-Intron structure was performed using Gene Structure Display Server (GSDS, http://gsds.cbi.pku.edu.cn/). Blue boxes represent UTR, red boxes represent exon, and black lines represent intron. (C) The conserved motifs of Si transporter homologous proteins in tomato. Ten conserved motifs (1-10) were indicated by different colors. The length of protein can be estimated using the scale at the bottom.
